# Undernutrition and associated factors among pregnant women in East Borena Zone, Liban District, Oromia regional state, Ethiopia

**DOI:** 10.3389/fnut.2022.1008701

**Published:** 2022-12-16

**Authors:** Godana Arero

**Affiliations:** Oromia Regional Health Bureau, Addis Ababa, Ethiopia

**Keywords:** undernutrition, wasting, stunting, underweight, anthropometric, MUAC, FFQ

## Abstract

**Background:**

Undernutrition is cellular imbalance between supply of nutrients, energy and body’s demand to ensure growth, maintenance, and specific function. However, there was no study conducted earlier on this topic in East Borena Zone.

**Objective:**

To assess the prevalence of undernutrition and associated factors among pregnant women in East Borena Zone, Liban District.

**Method:**

A community-based cross-sectional study was conducted on 420 study participants from November 20 to December 2021. The systematic sampling technique and simple random sampling methods were used to select study participants. Data were double entered into Epi-info software version 7 and SPSS version 21 software for analysis. Descriptive statistics were used to describe the characteristics of study participants. Bivariate and multivariable logistic regressions were carried out to identify the association between independent and dependent variables by measuring the adjusted odds ratio and 95% confidence interval. P-values less than 0.05 were considered statistically significant.

**Results:**

Prevalence of undernutrition among pregnant women was about (44.9%) of family monthly income [AOR = 8.72 (4.80, 15.83)], women’s decision-making autonomy [AOR = 0.40 (0.19, 0.82)], skipping meal [AOR = 2.62 (1.41, 4.89)], substance use [AOR = 2.01 (1.07, 3.77)], household food insecurity [AOR = 2.01 (1.06, 3.80)], lack of prenatal dietary advice [AOR = 2.73 (1.53, 4.89)], absence of household latrine [AOR = 9.23 (3.48, 24.46)], not participating health development army’s meeting at village level [AOR = 3.01 (1.57, 5.72)] and hand washing habit [AOR = 6.55 (3.02, 14.20)] had shown statistically significant association with undernutrition.

**Conclusion:**

The prevalence of undernutrition among pregnant women was high income. Women’s decision-making autonomy, skipping meals, substances use, household food insecurity, lack of prenatal dietary advice, poor hand washing habit, lack household of latrine, and not participation in health development army’s meeting were found to be predictors of the undernutrition.

## Introduction

Globally, undernutrition is an important health concern, predominantly in under-five children and pregnant women. The World Health Organization (WHO) classifies undernutrition as the greatest threat to public health (1) and every country is facing a serious challenge from undernutrition (2, 3). In spite of extensive global economic growth in recent decades, maternal undernutrition is highly prevalent in most countries in south-central and south-eastern Asia and Sub-Saharan Africa (4–6).

Ethiopia is one of the countries with a high burden of maternal and child undernutrition. Though maternal undernutrition has declined over the past 16 years, from 30% in 2000 to 22% in 2016, Ethiopia is still among the countries with a high burden of maternal undernutrition (7). Specifically, two institution-based cross-sectional studies conducted in the Amhara region reported a prevalence rate of undernutrition ranging from 16 to 29.8% (7).

Maternal undernutrition in low and middle-income countries is an underlining cause of 3.5 million mothers’ deaths and disabilities due to the physical and mental effects of poor dietary intake in the earliest months of life (8, 9). Previous studies have established that undernourished pregnant women suffer from a combination of chronic energy deficiency that leads them to have a low birth weight (LBW), and preterm and unsuccessful birth outcomes (10–12). Regardless of significant gains and signs of progress in the last decade, maternal undernutrition still remains a major public health problem in Ethiopia (13). The government of Ethiopia has developed a revised national nutrition program in 2016 to address the double burden of undernutrition in pregnant and lactating women (14). Even though the progress of this program implementation needs to be supported with a piece of continuous evidence through research, limited institution-based studies that lack an important variable crucial for prioritizing, designing, and initiating intervention programs have been conducted (15, 16).

Maternal undernutrition is highly prevalent in low and middle-income countries (17) and Ethiopia as one of these countries has been significantly affected by the burden of undernutrition. Many African women consume less than the recommended daily caloric intake and 5–20% are underweight. Pregnant women in industrialized countries gain on average twice as much as pregnant women in Africa. In 12/17 African countries, 10% or more of babies are born with low birth weight. Inadequate micronutrient intake, particularly of iron, vitamin A, zinc, folic acid, riboflavin, iodine, and vitamin E (18). Socio-demographic, economic, reproductive, medical, behavioral, healthcare, environmental, and dietary factors are associated with a pregnant mother’s nutritional status. Associated factors should be included in this research on page 18. A comparative study on maternal nutritional status in 16 of the 18 DHS-conducted countries and a study in the Oromia regional state showed that rural women are more likely to suffer from chronic energy deficiency than women in urban areas (17, 19). Dietary characteristics of the study participants such as (Minimum Dietary Diversity of Women, household food insecurity, improved dietary feeding, skipping meals/snacks, and eating additional meals were considered as associated with the nutritional status of pregnant mothers (20). The nutritional status of women before and during pregnancy can be determined by maternal knowledge, attitudes, and perceptions toward certain foods (21). Pregnancy is the most crucial nutritionally demanding period of every woman’s life. Appropriate nutrient intake during this period has a critical role in fetal development (22). Failure to receive necessary micro and macronutrient during this period will result in undernutrition and adverse pregnancy outcome (6). Undernutrition during pregnancy wields both short and long-term effect on the health of an infant by programming the infant’s development, increases the risk of non-communicable diseases, and is intimately related to the survival of both mothers and their babies (23). Hence, nutrition interventions such as nutrition education in different villages, health centres, health posts, and women’s organizations should be given to the community particularly for pregnant mothers concerning nutrition during pregnancy in the study area (24). At the national level, unmarried women were about 1.9 times more likely to be undernourished than currently married women, and the difference was statistically significant (25). Household economic status is one of the most important determinants of nutritional status in Ethiopian women. The study shows that, as compared with women residing in medium/higher economic status households, the risk of being undernourished for women in very poor or poor households was significant. This finding is consistent with other studies and the UNICEF conceptual framework (26, 27).

Women’s employment status is also another important socioeconomic variable explaining nutritional status. According to the findings of this study, unemployment or unpaid (cash) employment of women is a significant factor for chronic energy deficiency (CED) in these women as compared with women employed for cash. Women’s paid employment could provide an additional income source that can improve the food security of the household and raise the status of women by allowing them to have more control over resources. Some evidence also indicates that the nutritional impact of increased household income is a function of the income earner and the kind of income (10). It was also found that unemployed women were at high risk of undernutrition, even in households with a relatively better socioeconomic status (28). The objective of this study was, therefore, to assess the magnitude of undernutrition at the community level by including an important variables among pregnant women living in East Borena Zone.

**FIGURE 1:**
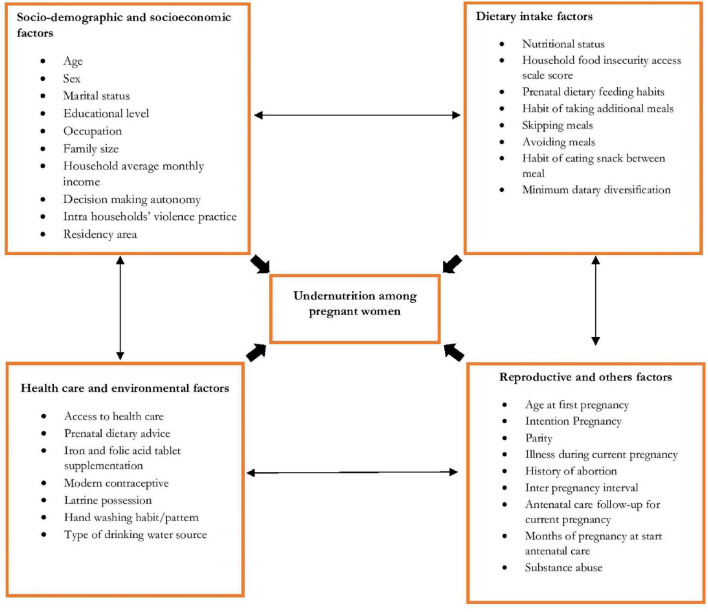
Conceptual framework assessing nutritional status among pregnant women in East Borena Zone, Liban District.

The conceptual framework used for this study was adopted and modified from UNICEF’s conceptual framework on the determinants of malnutrition (UNICEF (29). Maternal health status is greatly influenced by the dietary diversity and morbidity/physiological status of the mother which are the immediate causes (Figure 1). When the dietary diversity is poor it affects the woman’s morbidity status as there is reduced immunity and increased chances of developing infections. On the other hand, morbidity status in pregnancy affects dietary diversity either due to poor appetite which leads to only likable foods being selected, or some practices such as pica which affect nutrient intake.

## Materials and methods

### Study setting

The study was conducted in the East Borena Zone, Liban District, Oromia regional state in Ethiopia from November 20 to December 2021. The 2007 national census reported that the total population for this district was 138,813, of which 70,130 were men and 68,683 were women; 1,385 or 1% of that population were urban dwellers. During the study period, there were 22,081.

### Study period

It was conducted from November 20 to December 2021. Study design a community-based cross-sectional study design was conducted.

### Population

#### Source population

All pregnant women in the Liban District living in east Borena Zone for more than 6 months.

#### Study population

All randomly selected pregnant women living in the district during the study period.

### Inclusion and exclusion criteria

All pregnant women who lived in the district for more than 6 months, if chosen, were not included in the study, as were non-pregnant women who were severely ill.

### Sample size calculation and sampling techniques

The sample size was estimated using a single population proportion formula, considering 46.5% of the prevalence of undernutrition among pregnant women from a study conducted in Jimma Town (15).

Other parameters considered were 5% margin of error, 95%CI, and 10%, non-response rate.


$$\begin{array}{c} n=\frac{3.84\times 0.465\,\left(1-0.535\right)}{2}=382\\ d^{2}=\left(0.05*0.05\right) \end{array}$$

Where *n* = sample size Za = *Z*, value corresponding to a 95% level of significance = 1.96.

*p* = expected proportion of practices of mothers on nutrition during pregnancy = 50% = 0.5.

*d* = absolute precision (5%). Therefore, from the above, the sample size is = 382.

*n* = 382_10% none-response rate = 38, *n* = 382 + 38 = 420.

### Sample size determination for the second objective

The sample size for the second objective was calculated using the double population proportion formula using the Stat-calc of Epi-Info Statistical Software Version 7.0 with the following assumptions: Confidence level = 95%, power = 80%. The maximum sample size that was obtained from the general/first objective was 420. This sample was the larger sample size than the sample size calculated from the second specific objective. As a result, the final sample size decided for this study was 420, as determined by the first objective (Table 1).

**TABLE 1:** Sample size calculation for the second specific objective.

Variables	Magnitude		Power CI level	AOR	Sample size	References
					
	Exposed	Non exposed				
Average monthly income of HH	15.83%	4.8%	80%, 95%	8.72	73	(11)
Decision making autonomy of pregnant women	11.09%	1.81%	80%, 95%	2.7	160	(24)
Work load on women	28.8%	6.3%	80%, 95%	13.6	46	(24)
Frequent hand washing habits	14.20%	3.02%	80%, 95%	6.55	68	(11)
Educational status	2.91	0.77	80%, 95%	1.50	131	(7, 10)

### Sampling procedures

First, Kebeles (the lowest governmental administrative structure in Ethiopia) were stratified into urban and rural areas. The sample size was proportionally allocated for each stratum and then, representative pregnant women were randomly selected. A random sampling technique was utilized to select 10 Kebeles out of 35 total Kebeles. Finally, 420 samples were allocated proportionally to each selected Kebeles based on their total number of pregnant mothers. The calculated sample size of 420 study participants was proportionally allocated to randomly selected five health post out of 20 health post in the Liban District and two health centers out of five based on the number of clients attending antenatal care at health post and health center. Then, at each antenatal care unit, every seven pregnant women who were registered were included in the study until the desired sample size was reached.

The dependent variable of this study was the nutritional status of pregnant women. Independent variables were socio-demographic characteristics of the pregnant women included age, marital status, education, religion, ethnicity, residence area, family size, income, women’s decision-making, autonomy, intra-households violence, and polygamy. Reproductive, medical, and behavioral characteristics of the study participants like age at first marriage/pregnancy, trimester of pregnancy, pregnancy intention, gravidity, parity, abortion, inter-pregnancy interval, and recent illness in the past 15 days and substance abuse were independent variables considered in this study. Others were healthcare, and environmental characteristics such as accessibility to healthcare, prenatal dietary advice, antenatal care follow-up, drinking water source, and latrine possession. Dietary characteristics of the study participants such as minimum dietary diversity of women, household food insecurity, improved dietary feeding, skipping meals/snacks, and eating an additional meal are also independent variables included in the study.

### Data collection tools

During data collection, face-to-face interview, observation, anthropometric measurements, and standard structure questionnaires were used to collect data from pregnant women after the interviewers explained the purpose of the study and obtained the participant’s verbal consent to participate in the study. In this study, minimum dietary diversity for women (MDD-W) was measured by the Food and Agriculture Organization (FAO) (30) standard questionnaire developed for this purpose which is recommended for 24-h dietary recall.

Household food insecurity was measured by the Food and Nutrition Technical Assistance [FANTA (31)] standard tool that has nine questions each comprising three responses, 27-score-based Household Food Insecurity Access Scale (HFIAS) scale. The mid-upper arm circumference was used to assess malnutrition. MUAC [in cm] on their left arm at the midpoint between the tip of the shoulder (olecranon process) and the tip of the elbow (acromion process), and the insertion type of MUAC tape was benonelastic and non-stretchable to take with correct tension (not too loose/tight) with nearest 0.1 cm reading.

Age at pregnancy and afterbirth time were estimated using local memorable events. The participants were asked to recall and describe all the food and drink consumed in the previous 24 h from waking to sleeping. A recall interview contains 28 min to complete. In addition, anthropometric assessment mid-upper arm circumference (MUAC) measurement was involved. A structured questionnaire was developed and adopted from the Ethiopia Mini Demographic and Health Survey (EMDHS) (32) (EMDHS), the food frequency questionnaire, and the WHO standard. All the variables to be assessed were incorporated (Figure 1).

### Data quality assurance

For data quality control, study instruments were translated into local languages (by native speakers and then back translated to English by two other competent persons. Six interviewers and two supervisors were recruited for the survey and were trained on the overall data collection process. The six data collectors were BSC midwives and the supervisors were senior public health experts with Master’s degrees in local languages. Completeness and consistency of the data were assured through direct and daily supervision by the supervisors and principal investigator. Interviewers re-administered the questionnaires to the respondent under supervision by the supervisor. To ensure the quality of data, data collectors and supervisors were trained, and the questionnaire was translated into respondents’ native language to aid comprehension. In addition to written documentation of responses from study participants, tape recordings were done after obtaining verbal consent to ensure that all feedback were captured for analysis. Data collectors and supervisors were selected based on their educational background (particularly those who have received training on essential nutrition actions), work position, and experience in data collection. Supervisors and data collectors were trained on the objectives, methods, and data collection techniques of the study. Daily discussions and check-ups of data completeness were made with supervisors and the principal investigator. The data cleaning and entry were conducted exclusively by the principal investigator. The questionnaire was pretested among 5% of the total sample size to assess its clarity, length, completeness, and consistency. After the pretest was conducted, adjustments were done according to enhance the reliability and validity of the tool. The structured questionnaire was then rephrased in light of the responses. Test–retest reliability was established by examining the consistency of pretest responses using, and the three main components of the test–retest method are as follows: test–retest reliability of the research instrument was established during pretesting. Pretesting was done on two occasions but on the same respondents, on Monday and Friday: assume there is no change in the underlying condition (or trait you were trying to measure) between test 1 and test 2. Finally, compute the correlation between the two separate measurements; if test 1 and test 2 become consistent, the questionnaire was considered reliable.

### Statistical analysis

The collected data were checked for incompleteness and inconsistency. Data were entered into Epi-Info version 7.2 software and then exported to SPSS version 21 for analysis.

Prior to running for analysis, data were cleaned, composite indexes were computed and recorded over missing values, and extreme values were identified and trimmed. Descriptive statistics were used to describe the sample accordingly. Bivariate logistic regression was carried out to assess the association of each independent variable with acute undernutrition and those with *p*-values less than 0.25 remained in the final model (multivariate logistic regression). Odds ratios (ORs) were generated for each variable and the independence of any association was controlled by entering all variables into the model using the backward stepwise method. The magnitude of the association between the independent variables in relation to acute undernutrition was measured using adjusted odds ratios (AORs) and 95% confidence interval (CI) and *p*-values below 0.05 were considered statistically significant. Descriptive statistics were used to show socio-demographic characteristics and the prevalence of nutritional status. Logistic regression analysis was used to identify the association between factors, and the nutritional status of pregnant mothers and multivariate logistic regressions were performed to determine independent predictors of the nutritional status of pregnant mothers. A *p*-value < 0.05 was declared as statistically significant. The VIF and tolerance taste were checked for the presence of multicollinearity among the independent variables. The stepwise model building strategy with *p*-value = 0.05 was applied to identify independent predictors of nutritional status, and the Hosmer–Lemeshow test of goodness-of-fit was used to test how well the model explains the data. Adjusted odds ratios and their 95% confidence intervals were reported. Additionally, tables and figures were used to present the findings.

## Results

A total number of 420 pregnant women were interviewed making a response rate of 97.3%. The median ages of the mothers were (18.0 ± 1.2) years. The majority of the respondents were married (96.2%), housewives (75.5%), and residents of rural (78.8%). The overall prevalence of undernutrition among pregnant women was 44.9% [95%CI: (41.5, 50.1)] (Table 2).

**TABLE 2:** Socio-demographic, and socio-economic characteristics of study participants (*n* = 420), 2021.

Characteristics	Categories	Frequency	Percentage (%)
Residence area	Rural	309	78.8
	Urban	83	21.2
Age category in years	±25	109	27.8
	26–29	96	24.5
	30–33	102	26.0
	≥34	85	21.7
Current marital status	Single	3	0.8
	Married	377	96.2
	Widowed	10	2.6
	Other	2	0.5
Educational level	No formal education	110	28.1
	Primary education	144	36.7
	Secondary education	91	23.2
	Diploma and above	47	12.0
Occupation	Employee	11	2.8
	Private business	15	3.8
	Daily labourer	70	17.9
	Housewife	296	75.5
Family size	≤3	127	32.4
	4–6	189	48.2
	=7	76	19.4
Household average monthly income	≤2,000	126	32.1
	2,001–2,300	78	19.9
	2,301–3,000	117	29.8
	≥3,001	71	18.1
Decision making autonomy	Low	19	4.8
	Medium	166	42.3
	High	207	52.8
	Media	392	100.0
Intra households’ violence practice	No	342	87.2
	Yes	50	12.8

### Reproductive, medical, and behavioral characteristics of respondents

About 90% of pregnancies of women were planned and wanted. Around 85% of women do not have a history of illness, 93% had a history of abortion, 79% worked all household jobs alone, and 94.1% had substance abuse. Dietary characteristics of respondents The prevalence of undernutrition among pregnant women was (44.9%) and the rest were normal nutritional status. The household food security score indicates that 103 (26.5%) households were food insecure and 288 (73.5%) were food insecure. Out of the 391 respondents, 272 (69.4%) consumed six or more diets in the past 24 h, indicating that they had good minimum dietary diversification. More than three-quarters of pregnant women, 312 (79.6%), had a habit of taking additional meals, and 298 (76%) did not skip meals on a regular basis. Of the 420 study participants, 372 (98.2%) had consumed cereals in the previous 24 h. The main cereal consumed was teff in the form of injera/a staple food for Ethiopians made up of teff and certain barley and maize in the area. Vegetables held an integral part of the main meal for the majority of the study participants. More than 171 (43%) consumed vegetables, 123 (84.8%) consumed dark green leafy vegetables, and 124 (32.7%) consumed other vegetables. Oil and fat consumption was reported by 300 (76.5%) of the study participants. The white tubers and roots were consumed by 58 (14.8%; Table 3).

**TABLE 3:** Reproductive, medical, and behavioral characteristics of pregnant women in Liban District (*n* = 420), 2021.

Characteristics	Categories	Frequency	Percentage (%)
Age at first pregnancy (in year)	≤18	98	25.0
	19–20	155	39.5
	≥21	139	35.5
Intention pregnancy	Not planned and wanted	40	10.2
	Planned and wanted	352	89.8
Parity	≤2	214	54.6
	3–4	124	31.6
	≥5	54	13.8
Any illness during current pregnancy	Yes	59	15.1
	No	333	84.9
History abortion any type	No	365	93.1
	Yes	27	6.9
Inter pregnancy interval	≤18	119	30.4
	19–24	125	31.9
	25–28	60	15.3
	≥29	88	22.4
Antenatal care follow-up for current pregnancy	No	127	32.4
	Yes	265	67.6
Months of pregnancy at start antenatal care	≤4	239	61.0
	≥5	153	39.0
Working all household duties alone	No	310	79.1
	Yes	58	14.8
Substance abuse	Substance abused	22	5.6
	No substance abuse	369	94.1

### Dietary characteristics of respondents

The prevalence of undernutrition among pregnant women was (44.9%) and the rest were normal nutritional status. The household food security score indicates that 103 (26.5%) households were food insecure and 288 (73.5%) were food insecure. Out of the 391 respondents, 272 (69.4%) consumed six or more diets in the past 24 h, indicating that they had good minimum dietary diversification. More than three-quarters of pregnant women, 312 (79.6%), had a habit of taking additional meals, and 298 (76%) did not skip meals on a regular basis. Of the 420 study participants, 372 (98.2%) had consumed cereals in the previous 24 h. The main cereal consumed was teff in the form of injera/a staple food for Ethiopians made up of teff and certain barley and maize in the area. Vegetables held an integral part of the main meal for the majority of the study participants. More than 171 (43%) consumed vegetables, 123 (84.8%) consumed dark green leafy vegetables, and 124 (32.7%) consumed other vegetables. Oil and fat consumption was reported by 300 (76.5%) of the study participants. The white tubers and roots were consumed by 58 (14.8%; Table 4).

**TABLE 4:** Dietary characteristics of pregnant women living in Liban District (*n* = 420), 2021.

Characteristics	Categories	Frequency	Percentage (%)
Nutritional status	Under-nourished	30	7.7
	Normal	361	92.3
HFIAS score	Food insecure (<5)	103	26.5
	Food secure (≥5)	288	73.5
Prenatal dietary feeding habits	Unimproved	283	72.2
	Improved	109	27.8
Habit of taking additional meals	Yes	312	79.6
	No	79	20.2
Do you ever skip meals during this pregnancy	No	298	76.0
	Yes	94	24.0
Avoiding any food	No	256	65.3
	Yes	111	28.3
Habit of eating snack between meal	No	320	81.6
	Yes	33	8.4
Minimum datary diversification for women	≥6	272	69.4
	<6	120	30.6

A total of 98.2 present (*n* = 372) of the study population had consumed cereals in the previous 24 h which is predominant. The main cereal consumed was teffs in the form of Injeras which is a made of teff and certain barley and maze in the area. Vegetables form an integral part of the main meal for majority of the population generally. Over 43% (*n* = 171) consume vegetables; with 84.8% (*n* = 123) consuming dark green leafy vegetables and 32.7% (*n* = 124) consuming other vegetables. Oils and fats consumption was reported by 76.5% (*n* = 300) of the population. White tubers and roots were consumed by 14.8% (*n* = 58; Table 4). During the women affirmed that: “Most people consume cereal. This is what is easily available but those who have money may eat some meat but it depends on an individual’s economic ability.”

### Healthcare and environmental characteristics of respondents

Around 375 (95.7%) pregnant women had access to healthcare services traveling on foot for less than 1 h pregnant women 143 (79%) were supplied with iron–folic acid tablets during the second and third trimesters of pregnancy. About 270 (70.7%) had a history of using modern contraceptives for at least 1 year. There were 219 (55.9%) pregnant women who used latrines and washed their hands frequently (Table 5).

**TABLE 5:** Health care and environmental factors of pregnant women in Liban District (*n* = 420), 2021.

Characteristics	Categories	Frequency	Percentage (%)
Access to health care	No	17	4.3
	Yes	375	95.7
Prenatal dietary advice	No	356	90.8
	Yes	36	9.2
Iron and folic acid tablet supplementation	No	38	21
	Yes	143	79
Modern contraceptive	No	121	25.8
	Yes	270	70.7
Latrine possession	No	105	25.5
	Yes	287	71.7
Hand washing habit/Pattern	Not frequently	173	44.1
	Frequently	219	55.9
Type of drinking water source	Unprotected	85	21.7
	Protected	303	77.3

### Nutritional status and associated factors

The binary logistic regression analysis was performed for each variable written in the conceptual framework. Accordingly, average monthly income, decision-making autonomy, intrahousehold violence practice, history of any type of abortion, antenatal follow-up, current pregnancy intention, any illness during the current pregnancy, substance use (1 of these substances), household food security status, and type of latrine possessed were negatively associated with undernutrition. After binary logistic regression analysis, predictors with statistical significance and a *p*-value less than 0.25 were used in multivariable logistic regression analyses. In a multiple logistic regression analysis, those who had a gestational age between 25 and 28 months were 5.51 times more likely to be normal nutritional status compared to those who had gestational age above 33 months (AOR = 5.51, 95% CI: 1.27–23.96) and, those respondents who had been following antenatal care were six times more likely to be in normal nutritional status compared to those who had not been following ANC [AOR = 5.95, 95% CI: (1.49–23.82)] (see Table 6).

**TABLE 6:** Multivariate analysis test for nutritional status of pregnant women.

Characteristics	Nutritional status		*P*-value	Odds ratio (95%Cl)	
			
	Under-nutrition (MUAC < 23 cm)	Normal (MUAC ≥ 23 cm)		COR	AOR
**Household average monthly income in ETB**					
≤2,000	18 (14.3%)	108 (85.7%)	0.00	1	1
2,001–2,300	2 (2.6%)	76 (97.4%)	0.02	0.17 (0.04–0.77)*	0.30 (0.06–1.62)
2,301–3,000	8 (6.8%)	109 (93.2%	0.92	1.10 (0.15–8.03)	2.96 (0.31–28.18)
≥3,001	2 (2.8%)	69 (97.2%)	0.25	0.39 (0.08–1.91)	0.26 (0.05–1.55)
**Intra household violence practice**					
Yes	17 (34.0%)	33 (66.0%)	0.00	1	1
No	13 (3.8%)	329 (96.2%)	0.001	13.4 (5.82–29.19)**	18.81 (6.34–55.8)**
**Decision making autonomy**					
Low	4 (21.1%)	15 (78.9%)	0.04	1	1
Medium	13 (7.8%)	153 (92.2%)	0.03	0.25 (0.07–0.87)*	0.29 (0.05–1.82)
High	13 (6.3%)	194 (93.7%)	0.56	0.79 (0.36–1.75)	1.92 (0.62–5.95)
**Ant type of abortion**					
Yes	5 (18.5%)	22 (81.5%)	0.003	1	1
No	25 (6.8%)	340 (93.2%)	0.036	3.09 (1.08–8.86)*	4.30 (1.08–17.17)*
**This pregnancy intention**					
Planned	9 (22.5%)	31 (77.5%)	0.001	0.22 (0.09–0.52)**	0.29 (0.10–0.90)*
Unplanned	21 (6.0%)	331 (94%)	0.000	1	1
**Any illness during current pregnancy**					
Yes	9 (15.3%)	50 (84.7%)	0.000	1	1
No	21 (6.3%)	312 (93.7%)	0.021	0.37 (0.16–0.86)*	0.34 (0.10–1.09)
**Substance use (≥1 of these substances)**					
Yes	7 (31.8%)	15 (68.2%)	0.000	1	1
No	22 (6.0%)	347 (94%)	0.001	0.14 (0.05–0.37)**	0.16 (0.04–0.64)*
**Household food security status (HFIAS score)**					
Food secure	17 (5.9%)	271 (94.1%)	0.034	0.44 (0.21–0.94)*	0.65 (0.22–1.91)
Food insecure	13 (12.5%)	91 (87.5%)	0.000	1	1
**ANC follow up**					
Yes	26 (9.8%)	239 (90.2%)	0.012	3.35 (1.14–9.80)*	5.95 (1.49–23.82)*
No	4 (3.1%)	123 (96.9%)	0.0	1	1
**Type of latrine possessed**					
Improved	3 (2.8%)	105 (97.2%)	0.04	0.27 (0.08–0.92)*	0.22 (0.05–0.90)*
Unimproved	26 (9.5%)	246 (90.5%)	0.00	1	1
**Gestational age**					
≤24 months	4 (4.0%)	96 (96.0%)	0.53	1	1
25–28 months	9 (14.0%)	55 (56.0%)	0.02	1.92 (0.60–6.13)	5.51 (1.27–23.96)*
29–32 months	5 (7.60%)	61 (92.4%)	0.82	0.49 (0.20–1.22)	1.16 (0.32–4.41)
≥33 months	12 (7.4%)	150 (92.6%)	0.13	0.98 (0.33–2.88)	2.96 (0.74–11.78)

*Statistically significant at *P* value < 0.05. **Statistically significant at *P* value < 0.01. COR, crude odds ratio; AOR, adjusted odds ratio.

## Discussion

The study showed that (92.3%) of the women had normal nutrition status, while (44.9%) were undernourished. This finding is different from a study conducted in Nigeria which revealed the proportion of undernutrition among pregnant women was 11% (33). This might be because of socioeconomic status as Nigeria is richer than Ethiopia and the availability of infrastructure is better than health care delivery in our country. The finding of the present study is also higher than study conducted in Wolayita Sodo Town, high land of Ethiopia which showed overall, undernutrition status was 39.94% (95% CI: 34.7–45.2%), of which the majority (60%) had moderate anemia (33, 34). The reason behind the difference might be the present study was conducted in the most marginalized pastoralist area of the country where –frequent attacks of drought, shortage of rain, and above all conflict are more prevalent than in the highland area of the country. The majority of predictor factors of the present study like monthly income, decision-making autonomy, intra-household violence practice, history of any type of abortion, antenatal care follow-up, current pregnancy intention, any illness during the current pregnancy, substance use, food security, and type of latrine possessed were associated with undernutrition whereas in previous study family size >5 (AOR:7.74, 95%CI:4.15–16.47), multigravida (AOR:2.66, 95%CI:1.1.31–4.53), having a low income (AOR:5.81, 95%CI:2.93–14.11), current clinical illness (AOR: 6.38, 95%CI:3.13–13.00), intestinal parasitic infection (AOR:2.41, 95%CI:1.08–5.81), no history of contraceptive usage (AOR:5.02 95%CI:2.21–11.47), being in third trimesters (AOR:11.37, 95%CI:4.56–24.82, low body mass index (AOR:9.44, 95%CI:7.79–22.18) were identified as independent predictors of anemia among pregnant women (33, 34).

The prevalence of undernutrition in the present study is (44.9%) which was higher than a similar study conducted in Yemen, maternal under-nutrition was 11.4% (95% CI 3.8–35.2), maternal anemia (OR 5.3, 95% CI 1.5–18.6) and rural residents (OR 0.2, 95% CI 0.1–0.7) (35). This might be because of differences in socioeconomic status.

It is also higher than a study conducted in eastern Ethiopia which showed on average, 19.06% of subjects were malnourished, while 23.3% of study participants were underweight (body mass index < 19.8 kg m^–2^). In the final adjusted analysis, the risk of malnutrition was more than twofold higher in pregnant women with low (adjusted odds ratio = 2.47, 95% confidence interval = 1.41–4.34) and medium (adjusted odds ratio = 2.74, 95% confidence interval = 1.40–5.35) (23).

The present study finding is dissimilar from a study conducted in Shashemenne District, West Arsi Zone, South Ethiopia which revealed that 34.0% (95%CI: 29.5 and 38.4% of the women were undernourished (24). The possible reason as commonly mentioned the present study was conducted in a very low land area where agricultural production is very low and the food system always deteriorates by drought when compared to the previous study central Oromia.

The findings from present study were also highest when compare with different previous studies findings: For instance, study conducted among pregnant women visited Antenatal Care clinics in Silte zone, Southern Ethiopia showed: 21.8% (36); the study conducted in Southern Ethiopia among contextual risk factors for maternal malnutrition in a food-insecure zone revealed that 28.1% of the women were malnourished (BMI < 18.5) (37); study conducted among women in Addis Ababa, Ethiopia on The burden of underweight and overweigh was 21% (22); the study conducted in Konso district, Southern Ethiopia was the overall prevalence of undernutrition was 43.1% (95% CI 38.7–47.5%) (38); the prevalence of undernutrition among pregnant women in Gambella town was 28.6% (25); similar study conducted in Tigray Region Northern Ethiopia among pregnant women, revealed that the prevalence of undernutrition was found to be 40.6% with 95% confidence interval (38.93% and 42.27%) (39); the study conducted in Western Ethiopia showed that the magnitude of undernutrition among pregnant women was 39.2% (95%CI: 35.7, 42.6%) (40); the study conducted in in Eastern Ethiopia showed that the prevalence of undernutrition was 43.8% (95% confidence interval: 40.8, 47.0) (41); the study conducted in Gondar town, Northwest Ethiopia among pregnant mothers indicated that the prevalence of undernutrition was 14.4% (95%CI: 12.3–16.7) (42); the study conducted in Bench-Sheko and Kaffa zone, southwest Ethiopia among pregnant women showed that the prevalence of undernutrition was 23.7% (95% CI: 20.1, 27.4) (43); the study done in Nairobi, Kenya revealed that the prevalence of undernutrition among the pregnant women was 27% (44); the study conducted in Gedeo Zone, southern Ethiopia showed the prevalence of undernutrition among pregnant women was 21% (95% CI: 20.8–21.2) (45); the study done in Eastern Ethiopia revealed that the prevalence of undernutrition among pregnant was 43.8% (41). The study conducted in Uganda showed that the prevalence of underweight and stunting was 6.9% (318/4640) (46); the study conducted in Somali Region, East Ethiopia among pregnant women showed that the prevalence of undernutrition was 16.4% (47); the study done Somali region, Eastern Ethiopia revealed the prevalence of undernutrition was 35 (9%; 95%CI (30.8%, 40.2%) (48); the study conducted at Mettu Karl Referral Hospital, Southwest Ethiopia showed that the prevalence of undernutrition among pregnant was 17.5%. Family size > 5 [AOR = 8.2, 95%CI: 12.383, 46.217] (49).

The possible reasons for the difference might be in the Borena pastoralist zone shortages of rain, drought, and malnutrition are common epidemic problems. The Borena communities’ are many marginalized people in terms of primary health care delivery, safe dirking water, education, and the like. There is no almost food system people rely on for their daily energy intake. Livestock death is common because of drought. For this reason prevalence of malnutrition is expected to be higher than in other parts of the country.

The prevalence of the present study was lower than the study conducted in Ethiopia at large scale households among women’s malnutrition determinants which showed 59% was underweight (posterior odds ratio [OR] = 1.59; 95% credible interval [CrI]: 1.32–1.90). The possible reason might be the previous study used strong methods and Bayesian multilevel analysis. Furthermore, it was large-scale households along with a larger sample size compared to the present study (50).

The finding from the present study is slightly lower than (the 44.9%) study conducted in Ziway Dugda district, central Oromia 48.6% and 48.7% among pregnant women residing the in the Keserwan District in the Middle East (51, 52), respectively. The possible reason might be because of knowledge regarding utilization as the place where production is higher people use for commercials purposes rather than utilization at households and individual level and also health food eating is an issue to be considered.

The finding from the present study is lower than (44.9%), of a study conducted in Kacha Birra District, Southern Ethiopia which revealed (52.6%) of pregnant women were undernourished (53). The possible reason might be an epidemic outbreak (severe acute malnutrition) in Kacha Birra District increasing the proportion in the study area. However, it was higher than the study conducted in the same district Kacha Birra which showed the prevalence of anaemia among pregnant women was 29% (95% CI:1.18–5.84) (54) and also higher than the study conducted in Africa which indicated 23.5% (95%CI: 17.72–29.32; I2 = 98.5%) pregnant women were undernourished (7) and, also present study finding is higher than (44.9%), a study conducted in Miesso Health Centre in South Ethiopia among pregnant women indicated 30.3% was undernourished (55) and also study conducted in the pastoral communities of Afar Regional State in northeast Ethiopia was 30.9% [95% CI 26.5, 35.8%]. The possible reasons might be climate conditions, drought, and malnutrition in Borana Zone are more risk area than any part of Ethiopia.

The finding from the present study is higher than (44.9%), study conducted in Sudanese, Khartoum women who were underweight 4.4%), 95 (28.1%), 127 (37.6) reported (28). The possible reason might be the diagnostic method in the previous study was depend on blood peripheral and Haemoglobin tests which are preferable to simple MUAC, and BMI assessment methods.

## Conclusion

In this study, the prevalence of undernutrition among pregnant women was higher (44.9%) than in other similar studies. Average family income of households of the respondents, decision-making autonomy of pregnant women at the household level, Using Substance, household food insecurity, household average monthly income, women decision-making autonomy, intra-household violence practice during the current pregnancy, history of any type of abortion, gestational age, pregnancy intention, of any illness during current pregnancy and substance use, household food security status, pregnant women FANC attendant and household possessing improved type latrine were found to be independent predictors of pregnant mother nutritional status.

## Data availability statement

The original contributions presented in this study are included in the article/Supplementary material, further inquiries can be directed to the corresponding author.

## Ethics statement

The ethical approval was obtained from Oromia Regional Health Bureau Institutional review Board (IRB) depending on Helsiken ethical principles Ref. No. ORHB/1560/2021. Oral consent form was obtained from study participants.

## Author contributions

GA: conceptualization, proposal development, methodology, data collection, analysis, write-up, project administration, advising, consultation, revision, and edition.
