# Supplementary material for: Undernutrition and associated factors among pregnant women in East Borena Zone, Liban District, Oromia regional state, Ethiopia
Source: Front Nutr. 2022 Dec 16;9:1008701. doi: 10.3389/fnut.2022.1008701 (PMC9800510; doi:10.3389/fnut.2022.1008701)
Supplement: Supplementary file 1 [file Data_Sheet_1.pdf]

Supplementary Materials:

Supplementary Materials 1: English Version Questionnaire

Section 1: Questionnaire Identification Data

Code ______ Keble _________Worde (District) _____________Keble _________

Introduction:

My name is ------------------. I am working as data collector in a survey conducted by the Adam hospital medical college. We were interviewing women here about nutritional status and associated factors in order to generate information necessary for the planning of appropriate strategies (interventions) in the Lume woreda. To attain this purpose, your honest and genuine participation by responding to the question prepared is very important & highly appreciated.

Confidentiality and Consent:

We would like you to answer some personal questions that some people may find it difficult to answer. Your answers are completely confidential. Your name will not be written on this form. The nurses, doctors, and other people will not be told what you said in connection to your name. You do not have to answer any question if you don't want to and you can stop the interview at any time. However, your honest answer to these questions will help us to better understand the experience of mother's related nutritional status. We would greatly appreciate your help in responding to this study. The interview will take about 20 - 30 minutes. Would you be willing to participate?

If yes, proceed

If no, thank and stop here.

____

Supplementary Materials 2: A: Socio-Demographic Characteristics Pregnant Women in Lumen Woreda, East Shoa Zone, Oromia, Ethiopia 2019.

No	Questions	Coding categories	Skip
1.	How old are you? Maternal age in years (Probe for best estimate)	1. 15-24<br>2. 25-34<br>3. 35-45<br>4. ≥49	
2.	Where do live (Residence)?	1. Rural<br>2. Urban	
3.	What is the highest education level you completed?	1. No education<br>2. Primary<br>3. Secondary<br>4. Diploma and above	
4.	What is the highest education level you completed?	1. No education<br>2. Primary<br>3. Secondary<br>4. Diploma and above	
5.	What is your current marital status?	1. Orthodox<br>2. Protestant<br>3. Muslim<br>4. Catholic	
6.	What is your religion?	1. Orthodox<br>2. Protestant<br>3. Muslim<br>4. Catholic<br>5. Other	
7.	What ethnic or linguistic group do you belong to?		
8.	What is your current occupation?	1. Employee<br>2. Private business<br>3. Daily laborer<br>4. House wives<br>5. Other	
9.	What is your current occupation (husband)?	1. Employee<br>2. Private business<br>3. Daily laborer<br>4. Farmer<br>5. Other	

10.	What is your total monthly family income (approximately)?	1. 1000 Birr<br>2. 1000-2000 Birr<br>3. >=2000 Birr	
11.	Level of pregnant women decision-making autonomy.	1. Low<br>2. Middle<br>3. High	
12.	Do you have autonomy on health seeking?	1. Yes 0. No	
13.	Do you have autonomy on Major household purchases	1. Yes 0. No	
14.	Do you have autonomy on Major household expenditures?	1. Yes 0. No	
15.	Do you have autonomy on Visiting friends or participation on meetings?	1. Yes 0. No	
16.	Is there Intra-household violence on current pregnancy?	1. Yes 0. No	

Supplementary Materials 3: Reproductive Characteristics of Pregnant Women in Lumen Woreda, East Shoa Zone, Oromia, Ethiopia 2019.

No	Questions	Coding categories	Skip
17.	How old were you at first pregnancy.	1. 15-24<br>2. 25-34<br>3. 35-45<br>4. ≥49	
18.	How money alive children do you have currently________?	1. 0 (No Birth)<br>2. 1-3<br>3. ≥ 3	
19.	Is this your 1st pregnancy?	1. Yes 0. No	
20.	What is this intention you have on current Pregnancy?	1. Planned and wanted<br>0. Not planned and wanted	
21.	Are you following FANC?	1. Yes 0. No	
22.	How many months was your pregnancy when you start of ANC follow up _________________?	1. 1-3 months<br>2. 4-6 months<br>3. 7-9 months	

23.	How long is it since you become currently pregnant? ______in weeks<br>What is the trimester of the pregnancy?	1. First<br>2. Second<br>3. Third	
24.	How many pregnancies you ever have?	1. 0 (No pregnancy)<br>2. 1-3<br>3. ≥ 3	
25.	Did you ever have history of abortion of any type?	1. Yes 0. No	
26.	How long is it since you become pregnant currently?	0. < 28 weeks<br>1. ≥ 28 week	
27.	How long is interval between successive births	□ 0 (No birth)<br>□ ≥ 2years<br>□ < 2 years	

Supplementary Materials 4: Medical Behavioural Characteristics of Pregnant Women in Lumen Woreda, East Shoa Zone, Oromia, Ethiopia 2019.

No	Questions	Coding categories	Skip
28.	Did you experience illness in the past 15 days?		
29.	How do you feel about your weight change since you become pregnant?	1. Gaining too much<br>2. Gaining too little<br>3. Has no change<br>4. I'm not sure	

30.	Which of the following do you do?	1. Drink alcohol<br>2. Chew chat<br>3. Smock cigarettes<br>4. None of them	
31.	Have you ever used modern contraceptive of any type?	1. Yes 0. No	
32.	Do you have ITN at your home?	1. Yes 0. No	
33.	Do you sleep ITNS	1. Yes 0. No	
34.	Did you ever advised to eat more meals during this pregnancy?	1. Yes 0. No	
35.	Did you ever advised to eat balanced diet during this pregnancy?	1. Yes 0. No	
36.	Did you ever advised to eat fruits/vegetable during this pregnancy?	1. Yes 0. No	
37.	Do you have national family health guideline for current pregnantacy?	1. Have<br>2. No, I Have not	
38.	Have you been screened for wasting during current pregnancy?	1. Yes 0. No	
39.	Have you been Attended dietary feeding practice?	1. Yes 0. No	
40.	What time does it take to you to reach nearest health facility?	0. < One hr ≥ One hr	
41.	Do you have latrine?	1. Yes 0. No	
42.	What type of latrine do you have?	1. Improved<br>0. Unimproved	
43.	Do you wash your hand before and after required hand washing time?	0. Not frequently<br>1. Frequently	
44.	What source of water do you use for drinking?	0. Unprotected<br>1. Protected	

Supplementary Materials 5: Household Food Insecurity Access Scale (HFIAS) Measurement Tool

NB: 1. Rarely (once or twice in the past four weeks) 2. Sometimes (three to ten times in the past four weeks) 3. Often (more than ten times in the past four weeks)			
1	In the past four weeks, did you worry that your household would not have enough food?	1. Yes	2. No (skip to Q2)
1.1	How often did this happen? 1. Rarely	2.Sometimes	3.Often
2	In the past four weeks, were you or any household member not able to eat the kinds of foods you preferred because of a lack of resources?	1. Yes	2. No (skip to Q3)
2.1	How often did this happen? 1. Rarely	2.Sometimes	3.Often
3	In the past four weeks, did you or any household member have to eat a limited variety of foods due to a lack of resources?	1. Yes	2. No (skip to Q4)
3.1	How often did this happen? 1. Rarely	2.Sometimes	3.Often
4	In the past four weeks, did you or any household member have to eat some foods that you really did not want to eat because of a lack of resources	1. □ Yes	2. No (skip to Q5)
4.1	How often did this happen? 1. Rarely	2.Sometimes	3.Often
5	In the past four weeks, did you or any household member have to eat a smaller meal than you felt you needed because there was not enough food?	1. □ Yes	2. No (skip to Q6)
5.1	How often did this happen? 1. Rarely	2.Sometimes	3.Often
6	In the past four weeks, did you or any other household member have to eat fewer meals in a day because there was not enough food?	1. Yes	2. No (skip to Q7)
6.1	How often did this happen? 1. Rarely	2.Sometimes	3.Often
7	In the past four weeks, was there ever no food to eat of any kind in your household because of lack of resources to get food?	1. Yes	2. No (skip to Q8)
7.1	How often did this happen? 1. Rarely	2.Sometimes	3.Often
8	In the past four weeks, did you or any household member go to sleep at night hungry because there was not enough food?	1. Yes	2.No (skip to Q9)
8.1	How often did this happen? 1. Rarely	2.Sometimes	3.Often
9	In the past four weeks, did you or any household member go a whole day and night without eating anything because there was not enough food?	1. Yes	2.No (skip to Q9)
9.1	How often did this happen? 1. Rarely	2.Sometimes	3.Often

Supplementary Materials 6: Minimum Dietary Diversity for Women

Model list-based questionnaire. Yesterday during the day or at night, did you eat or drink:

SNo	Food categories	Description	Consumed Yes = 1, No = 0	
8.1	Any foods made from grains.	Did you eat foods made from grains, like: Enjera, Porridge, bread, rice, pasta/noodles or other foods made from grains?	1. Yes	0. No
8.2	Any vegetables or roots that are orange-coloured inside	Did you eat Any vegetables or roots that are orange-coloured inside, like: carrots, sweet potatoes that are yellow or orange inside?	1. Yes	0. No
8.3	Any white roots and tubers or plantains.	Did you eat potatoes,	1. Yes	0. No
8.4	Any dark green leafy vegetables.	Did you eat dark green leafy vegetables like cabbage?	1. Yes	0. No
8.5	Any fruits that are dark yellow or orange inside	Did you eat ripe mango, ripe papaya	1. Yes	0. No
8.6	Any meat made from animal organs, such as	Liver, kidney, heart or other organ meats or blood-based foods, including from wild game	1. Yes	0. No
8.7	Any other types of meat or poultry	lamb, goat, chicken, other	1. Yes	0. No
8.8	Any eggs	Eggs from poultry or any other bird	1. Yes	0. No
8.9	Any fish or seafood, whether fresh or dried	Fresh or dried fish, shellfish or seafood	1. Yes	0. No
8.10	Any beans or peas	Mature beans or peas (fresh or dried seed), lentils or bean/ pea products	1. Yes	0. No
8.11	Any nuts or seeds,	Any tree nut, groundnut/peanut, or certain seeds or nut/seed "butters" or pastes	1. Yes	0. No
8.12	Any milk or milk products	Milk, cheese, yoghurt or other milk products, but NOT including butter, ice cream, cream or sour cream	1. Yes	0. No
9	Household food insecurity	What does your household food security look like?	1.Secured	0.Insecure

Supplementary 7: Assurance of Principal Investigator

The undersigned agrees to accept responsibility for the scientific ethical and technical conduct of the research project and for provision of required progress reports as per terms and conditions of the Research Publications Office in effect at the time of Grant is forwarded as the result of this application.

Name of the student: ____

Date. _____________________________ Signature ________________

Approval Form of Advisors:

Name of the primary advisor: ____________________________________

Signature ___________________________ Date. ____________________

Name of the secondary advisor: __________________________________

Signature ___________________________ Date. ____________________
